# Vitamin A Is a Negative Regulator of Osteoblast Mineralization

**DOI:** 10.1371/journal.pone.0082388

**Published:** 2013-12-10

**Authors:** Thomas Lind, Anders Sundqvist, Lijuan Hu, Gunnar Pejler, Göran Andersson, Annica Jacobson, Håkan Melhus

**Affiliations:** 1 Department of Medical Sciences, Section of Clinical Pharmacology, Uppsala University, Uppsala, Sweden; 2 Department of Anatomy, Physiology and Biochemistry, Swedish University of Agricultural Sciences, Uppsala, Sweden; 3 Division of Pathology, Department of Laboratory Medicine, Karolinska Institute, Karolinska University Hospital, Huddinge, Sweden; INSERM U1059/LBTO, Université Jean Monnet, France

## Abstract

An excessive intake of vitamin A has been associated with an increased risk of fractures in humans. In animals, a high vitamin A intake leads to a reduction of long bone diameter and spontaneous fractures. Studies in rodents indicate that the bone thinning is due to increased periosteal bone resorption and reduced radial growth. Whether the latter is a consequence of direct effects on bone or indirect effects on appetite and general growth is unknown. In this study we therefore used pair-feeding and dynamic histomorphometry to investigate the direct effect of a high intake of vitamin A on bone formation in rats. Although there were no differences in body weight or femur length compared to controls, there was an approximately halved bone formation and mineral apposition rate at the femur diaphysis of rats fed vitamin A. To try to clarify the mechanism(s) behind this reduction, we treated primary human osteoblasts and a murine preosteoblastic cell line (MC3T3-E1) with the active metabolite of vitamin A; retinoic acid (RA), a retinoic acid receptor (RAR) antagonist (AGN194310), and a Cyp26 inhibitor (R115866) which blocks endogenous RA catabolism. We found that RA, via RARs, suppressed *in vitro* mineralization. This was independent of a negative effect on osteoblast proliferation. Alkaline phosphatase and bone gamma carboxyglutamate protein (Bglap, Osteocalcin) were drastically reduced in RA treated cells and RA also reduced the protein levels of Runx2 and Osterix, key transcription factors for progression to a mature osteoblast. Normal osteoblast differentiation involved up regulation of Cyp26b1, the major enzyme responsible for RA degradation, suggesting that a drop in RA signaling is required for osteogenesis analogous to what has been found for chondrogenesis. In addition, RA decreased Phex, an osteoblast/osteocyte protein necessary for mineralization. Taken together, our data indicate that vitamin A is a negative regulator of osteoblast mineralization.

## Introduction

Excessive vitamin A (retinol) intake is a risk factor for fracture in humans and the vitamin is the only known compound that can induce spontaneous fractures of long bones in animals [1]-[4]. Studies in rodents have shown that these spontaneous fractures are caused by a reduced bone diameter, whereas there is little or no effect on bone mineral density [5]. This bone thinning, in turn, appears to be caused by increased periosteal bone resorption and reduced diaphyseal radial growth [6], [7]. However, since a high vitamin A intake also results in anorexia and reduced weight gain, it is unclear whether the observed reduction of bone formation is a direct effect of vitamin A on bone or a consequence of indirect, systemic effects on appetite and general growth. To date, there are no studies that have controlled for these indirect effects, nor are there studies that have included dynamic histomorphometry at the diaphyseal site of the long bones.

Except in the eye, retinol is converted to retinal and then to retinoic acid (RA) in target cells, where RA binds to specific nuclear RA receptors (RARs). RAR expression has been shown in both primary human osteoblasts and in the murine preosteoblastic cell line (MC3T3-E1) [8], [9]. The intracellular RA concentration is determined by the balance between the activity of aldehyde dehydrogenase driven RA synthesis and the RA-specific inactivation by the oxidizing P450 enzymes (CYP26 A, B and C). CYP26B1 expression has been shown to be increased by RA and reduced by a pan-RAR antagonist, indicating that this gene is a direct target of RA [10]–[12]. Human null and hypomorphic mutations in this major regulator of RA concentration in osteoblastic cells, CYP26B1, lead to severe skeletal anomalies, demonstrating the importance of strict regulation of intracellular RA levels also for human bone health [13].

Osteoblast differentiation is initiated by the expression of a key transcription factor named Runx2 in progenitor cells, leading to the generation of preosteoblasts. Runx2-deficient mice show a complete lack of ossified bones and, hence, Runx2 has been implicated as the master gene of osteoblast differentiation [14]. In preosteoblasts, Runx2 induces Sp7 (Osterix), which is an essential transcription factor for the initiation of extracellular matrix production and mineralization. The mature osteoblast is typically characterized by high bone gamma carboxyglutamate protein (Bglap, Osteocalcin) expression. As the osteoblasts become surrounded by mineralized bone they reach their differentiation endpoint, switching to an osteocyte phenotype, which is characterized by dentin matrix protein 1 (Dmp1), sclerostin (Sost) and fibroblast growth factor 23 (Fgf23) expression. Osteocytes, which make up more than 90% of all bone cells in the adult skeleton, were recently shown to be of major importance in regulating bone homeostasis by being the main source of the cytokine receptor activator of nuclear factor-κB ligand (RANKL, Tnfsf11) [15], [16]. The membrane-bound, full-length RANKL protein is considered the pivotal form, inducing osteoclastogenesis by binding to RANK on osteoclast progenitors [16]–[18].

Although there are numerous studies of RA effects in osteoblasts, information on the effects on bone formation *in vitro* is still very sparse and in a recent review it was concluded that “it is not possible to reach a firm conclusion regarding vitamin A action at this time.” [7]. The aim of this study was therefore to clarify the direct effect of vitamin A and its active metabolite RA on osteoblast mineralization, both *in vivo* and *in vitro*.

## Materials and Methods

### Animals

This study was carried out in strict accordance with the recommendations in the Guide for the Care and Use of Laboratory Animals of Sweden. The protocol was approved by the Committee on the Ethics of Animal Experiments of the University of Uppsala (Permit Number: C254/7). Male Sprague-Dawley rats, 5 weeks of age, were obtained from Möllegaards Breeding Centre, Ltd. (Skensved, Denmark). They were fed a standard diet (Lactamin R36, Stockholm, Sweden) containing 12 IU vitamin A/g pellet (“Control”), or a standard diet supplemented with 1700 IU vitamin A/g pellet (“Vitamin A”) for 7 days [19]. The control group is a pair-fed group, i.e. the control animals were fed the same amount of chow as consumed by the vitamin A group to keep food intake and body weight gain of the groups the same (n = 10/group). Vitamin A was added to the pellets in the form of retinyl palmitate and retinyl acetate. At the end of the experiment, the rats were sacrificed by exsanguinations from the abdominal aorta under Eqvitesin anesthesia (chloral hydrate 182 mg/kg, pentobarbital 41.7 g/kg) and all efforts were made to minimize suffering.

### Serum analyses

Serum analyses of vitamin A were done by AS Vitas (Oslo, Norway) on samples (n = 10/group) shielded from light. Briefly, 200 µL of serum or standard solutions was mixed with 600 µL of 2-propanol and centrifuged at 4000 g. The supernatant was analyzed by liquid chromatography on an HP-1100 HPLC system furnished with a Supersphere 100 RP-18 column (Agilent Technologies, Palo Alto, CA) and detected at 325 nm with an ultraviolet detector. Fgf23 (n = 10/group) was measured using the ELISA kit for rat FGF23 (Cusabio, Cat no. CSB-E12170r) and phosphate (n = 10/group) was quantified with the Quantichrome Phosphate Assay Kit (DIPI-500, Hayward, CA).

### Histomorphometry

Histomorphometric parameters were measured from the diaphysis (cortical bone) of the femur of four randomly selected animals per group at Pharmatest, Finland, as recommended by the American Society for Bone and Mineral Research Histomorphometry Nomenclature Committee [20]. The analysis of cortical bone was done using BioQuant Osteo II software version 8.12 (BioQuant Image Analysis Corporation, Nashville, TN). Bones were double-labeled with calcein at day 0 and at day 6 prior to the scheduled terminal necropsy at day 7 to measure dynamic parameters. The following parameters were determined at the periosteum: Total cross-sectional area of the bone (mm^2^), Mineralizing surface (%), Mineral apposition rate (µm/day) and Bone formation rate/bone surface (µm^3^/µm^2^/day).

### Reagents

Alizarin Red S, cetylpyridinium chloride and all-*trans*-retinoic acid (RA) were from Sigma-Aldrich, Sweden. RA was dissolved in 95% ethanol in a dark room under the flow of nitrogen. The 2.0 mM stock solution was shielded from light and stored at –70°C until use. The Cyp26 specific inhibitor, R115866 (talarozole, a gift from Barrier Therapeutics, Geel, Belgium) and the high affinity pan-RAR-antagonist (AGN194310, a gift from Dr. Chandraratna, Allergan Inc, Irvine, CA) were dissolved in dimethyl sulfoxide (DMSO). Primary antibodies were: mouse monoclonal anti-Runx2 (D130-3) (MBL, Japan), mouse monoclonal anti-ActB (sc47778), rabbit polyclonal anti-Osterix (sc-22536-R), goat polyclonal anti-RANKL (sc-7628), goat polyclonal anti-Phex (sc-47324) from Santa Cruz Biotech. (Santa Cruz, CA), rabbit polyclonal anti-Dmp1 (M176) (Takara Bio Inc., Japan) and a rabbit polyclonal anti-Cathepsin K [21].

### Cell culture

Primary human osteoblasts were isolated from bone obtained from male donors undergoing knee replacement surgery and had no reported bone-related pathologies other than osteoarthritis. The osteoblastic phenotype of cells was verified by use of biochemical markers as previously described [22]. Uppsala University Hospital ethics committee approved this study (Permit Number: Dnr Ups 03-561) and waived the need for consent from these de-identified donors. The primary human osteoblasts and the mouse preosteoblast cell line, MC3T3-E1 subclone 4 (from ATCC) were cultured in α-MEM supplemented with 10% heat inactivated fetal bovine serum, 2 mM L-glutamine, 100 µg/ml streptomycin and 100 U/ml penicillin. To induce osteoblastogenesis, cells were switched to osteogenic media (control) containing 25 µg/ml ascorbic acid and 10 mM β-glycerophosphate. Change of media was done every 2nd or 3rd day. At the end of the experiment total RNA was extracted using TRI Reagent® (Sigma-Aldrich) or protein was extracted using 2 x SDS-PAGE sample buffer according to the BioRad protocol.

### Cell proliferation

Mouse MC3T3-E1 cells were seeded in control medium with or without 400 nM RA for 10 days and with 400 nM RA or 1 µ RAR-antagonist (AGN) for 14 days. Cell proliferation was measured with a MTT kit (Sigma-Aldrich) in a 96-well plate and by cell number counting in a 12-well plate using NucleoCounter™ (Chemometec, Allerød, Denmark) according to the manufacturer’s instructions. Each experiment was performed at least three times using triplicates.

### Quantitative RT-PCR

Four hundred ng of total RNA was transcribed to cDNA using the TaqMan system (Applied Biosystems, USA). Quantitative real time PCR was performed using inventoried TaqMan® Gene Expression Assays for *Cyp26b1* ENSMUSG00000063415, (Mm00558507_m1), *Alpl* ENSMUSG00000028766, (Mm01187117_m1), *Bglap* (*Osteocalcin)* ENSMUSG00000074483 (Mm03413826_mH), *Runx2* ENSMUSG00000039153 (Mm00501580_m1), *Sp7* (*Osterix)* ENSMUSG00000060284 (Mm00504574_m1), *Phex* ENSMUSG00000057457 (Mm00448119_m1), *Dmp1* ENSMUSG00000029307 (Mm01208363_m1), *Sost* ENSMUSG00000001494 (Mm00470479_m1), *Tnfsf11* (*RANKL)* ENSMUSG00000022015 (Mm00441908_m1) and *Fgf23* ENSMUSG00000000182 (Mm00445621_m1) according to the manufacturer's protocol, on a TaqMan 7000 apparatus. Cycling protocol: 50°C for 2 min, followed by 95°C for 10 min and then 40 cycles of 95°C 15 sec followed by 60°C for 1 min. For standardization, expression levels were divided by expression level for *Actb* ENSMUSG00000029580 (Mm00607939_s1), derived from dilution standard curves of Ct values for each gene. Each experiment was performed at least three times using triplicates.

### Alizarin-S Red staining

Cells were rinsed 2 times with PBS, fixed in ice-cold 70% ethanol for 1h and then stained with 40 mM Alizarin-S Red (pH 4.2), for 10 min with shaking. The amount of stain was quantified by solubilization with 10% cetylpyridinium chloride followed by reading the absorbance at 560 nm. Each experiment was performed at least three times using quadruplicates.

### Immunoblotting

Cell lysates were boiled for 5 minutes and DNA was sheared with a 21G needle followed by protein determination using the BCA protein reagent (Pierce, Rockford, IL). An equal amount of protein was separated by SDS-PAGE as described previously [23]. The primary antibody was detected with a horseradish peroxidase conjugated secondary antibody (DAKO, Denmark), which was diluted 1∶20,000 and then processed by chemiluminescence with ECL reagents (Millipore, Billerica MA). The pixel density of the bands was assessed using ImageJ software (U.S. National Institutes of Health, Bethesda, MD, USA).

### Immunohistochemistry

The bone (humeri) preparation and immunohistochemistry have previously been described in detail [24]. The bones from all animals were sectioned in the same orientation in order to make comparable sections. Visualization of the primary antibodies where achieved by incubation with secondary biotinylated antibody at a dilution of 1∶200 in 10% serum and PBS followed by an avidin-biotin-peroxidase complex incubation using the Vectastain ABC-kit (Vector Laboratories) and the substrate diaminobenzidine tetrahydrochloride (DAB, DAKO).

### Statistical Analyses

The data were analyzed by the Students t-test or, for variables with deviations from the normal distribution, the Mann-Whitney U test. In every case, p<0.05 was considered statistically significant.

## Results

### A high dietary vitamin A intake leads to a reduced mineral apposition rate

**Figure 1:**
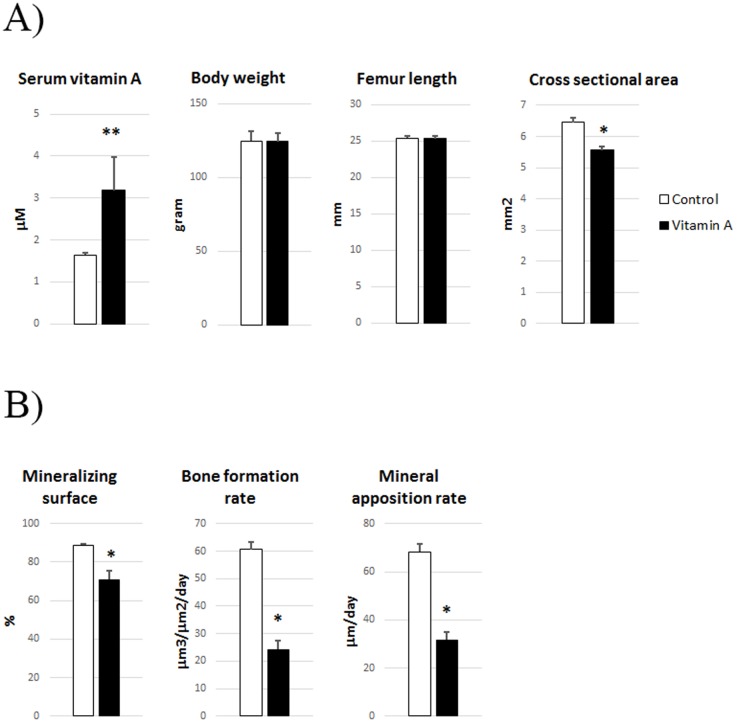
Effects of a high vitamin A intake on serum levels, bone and body weight. (**A**) Comparison of serum vitamin A levels, body weight, femur length and cross sectional area of mid diaphysis of femur, and (**B**) periosteal mineralizing surface, bone formation rate and mineral apposition rate as determined by histomorphometric analysis of calcein double-labeled bones in rats with a high vitamin A intake and pair-fed controls. Means +/– SEM, * p<0.05 vs Control.

To control for indirect effects of vitamin A on appetite and general growth, we applied pair-feeding, i. e. the control rats were fed the same amount of food as that consumed by the vitamin A group. The vitamin A group acquired an approximately doubling of vitamin A levels in serum (Fig. 1A). Although there were no differences in final body weight or femur length, rats with hypervitaminosis A had thinner bones as they evinced a diminished total cross-sectional bone area (12%) at the mid diaphysis of the femur (Fig.1A). Dynamic histomorphometric analysis of these bones clearly showed that a high intake of vitamin A reduced the mineralizing surface (20%), bone formation rate (60%) and the mineral apposition rate (54%) (Fig. 1B). These results indicate that a high vitamin A intake has direct and inhibiting effects on bone formation.

### Retinoic acid reduces osteoblast mineralization *in vitro*

**Figure 2:**
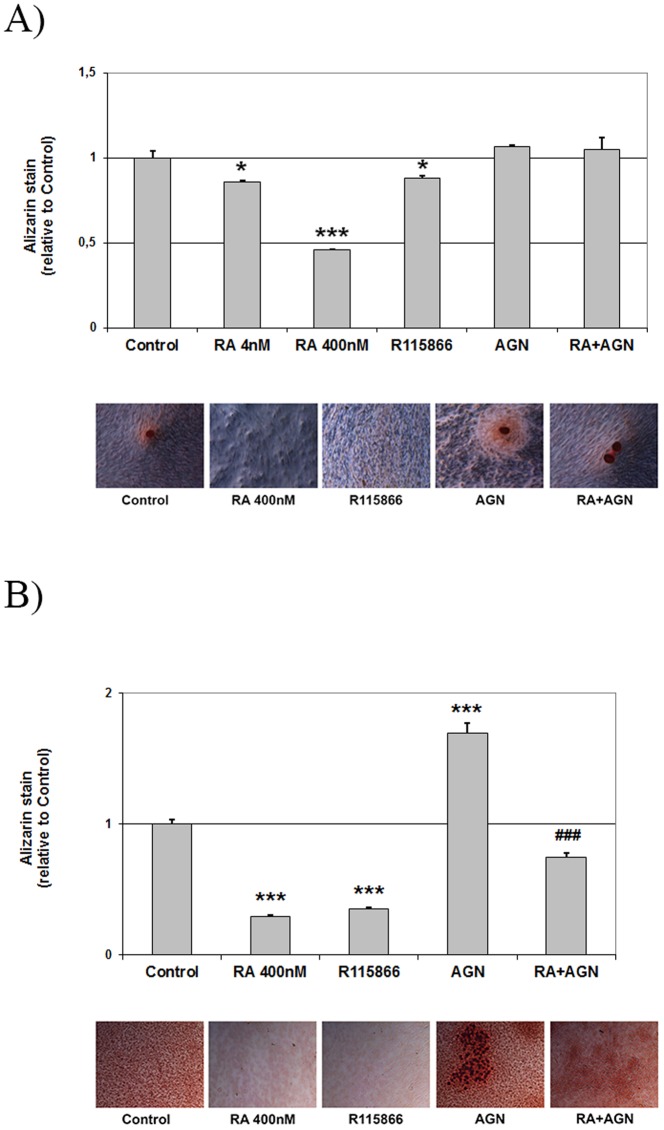
The effect of RA and RAR signaling on osteoblast mineralization *in vitro*. (**A**) Representative diagram of quantification of Alizarin Red stain in primary human osteoblasts (from a single individual) treated with RA at 4 and 400 nM, a Cyp26 inhibitor (R115866, 5 µM) and a pan-RAR antagonist (AGN, 1 µM) and 400 nM RA + AGN for 25 days. Below are representative photographs of the cultures. (**B**) Alizarin Red stain quantification of the mouse preosteoblast cell line MC3T3-E1 treated with RA at 400 nM, a Cyp26 inhibitor (R115866, 5 µM) and a pan-RAR antagonist (AGN, 1 µM) and 400 nM RA + AGN for 25 days. Below are representative photographs of the cultures. Means +/– SD, not significant (ns), * p<0.05 and *** p<0.001, vs Control, and ### p<0.001 vs RA.

To clarify the specific effect of vitamin A on osteoblasts, we first added RA to cultures of primary human osteoblasts. As seen in Figure 2A, addition of 4 nM or 400 nM RA reduced calcium deposition by 14 and 54%, respectively, as quantified by Alizarin Red staining. Increasing endogenous RA concentrations using an inhibitor of the intracellular RA-degrading Cyp26 enzymes (R115866), reduced osteoblast mineralization by 12%. Addition of a pan-RA receptor (RAR)-antagonist (AGN) induced a small, non-significant increase (7%) in Alizarin Red staining. As preparations of primary cells from bone may contain other cells types, we then performed mineralization experiments using the mouse osteoblast cell line MC3T3-E1. In these cells, the effects of RA, the Cyp26 inhibitor and the RAR antagonist were even more pronounced (Fig. 2B). Thus, addition of 400 nM RA or R115866 reduced Alizarin staining by 70 and 65%, respectively. AGN addition alone distinctly increased Alizarin staining to 170% of control. The significant increase in mineralization after adding the RAR antagonist indicates that the RA effect is RAR-dependent and that blocking endogenous RA is sufficient to increase mineralization. Concomitant addition of AGN completely reversed the effect of RA in the human cells and partly reversed the negative effect of RA on mineralization in the mouse cell line (Fig. 2A,B).

### The reduced mineralization is not only a consequence of inhibited cell proliferation

**Figure 3:**
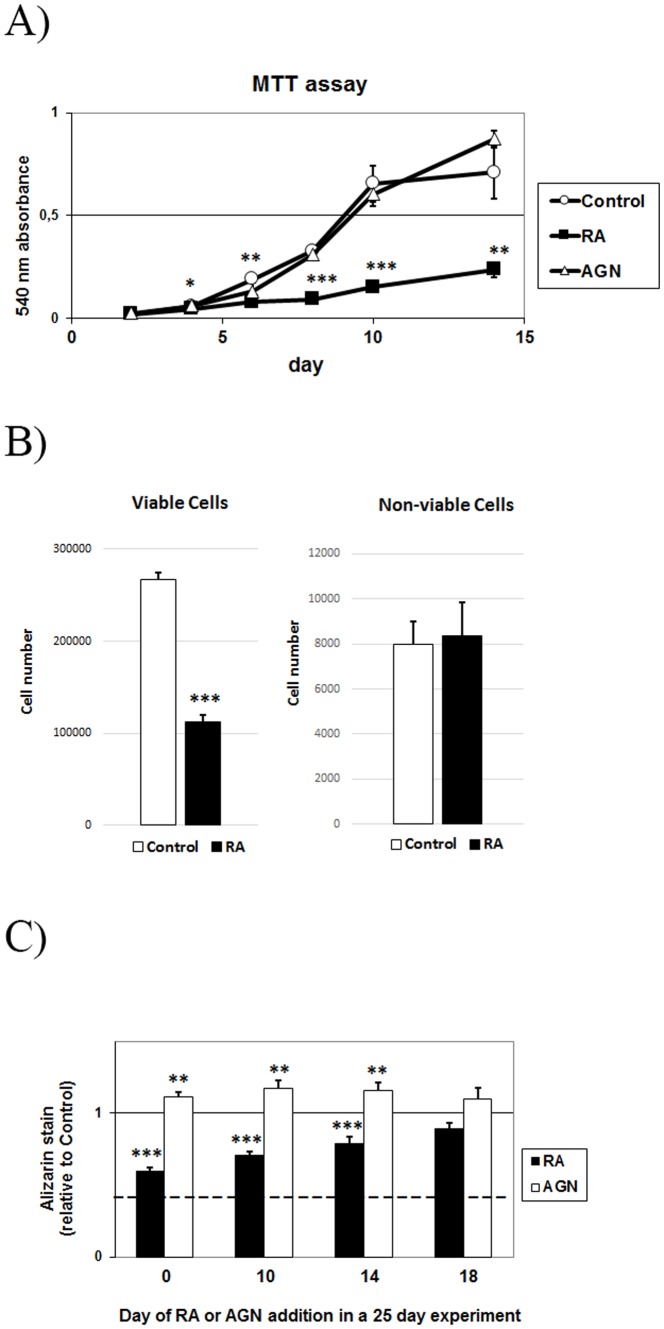
RA and RAR-dependent effects on osteoblast proliferation and on treatment start during *in vitro* mineralization. (**A**) Cell proliferation of MC3T3-E1 cells, treated with 400 nM RA or 1 µM AGN during the first 14 days of a mineralization experiment. (**B**) Cell number of viable and non-viable MC3T3-E1 cells after 10 days, with or without 400 nM RA. (**C**) Alizarin Red stain quantification of MC3T3-E1 cells, treated with 400 nM RA or 1 µM AGN from day 0, 10, 14 or 18 followed by analysis at day 25. Control mineralization level is set at 1 and dotted line represent background (no osteogenic induction). Means +/– SD, * p<0.05, ** p<0.01 and *** p<0.001 vs Control.

Next, we measured cell proliferation during MC3T3-E1 mineralization experiments using an MTT-based proliferation assay. As seen in Fig. 3A, RA inhibited MC3T3-E1 proliferation and the RAR-antagonist (AGN) did not produce any detectable difference compared to control cells. Control cell proliferation appears to level off after day 10. The inhibition of cell proliferation by RA was confirmed by counting cells grown with or without 400 nM RA for 10 days (Fig. 3B). The number of non-viable cells did not differ from control cultures (Fig. 3B). Next, to determine whether RA:s inhibitory effect on mineralization was dependent on its reduction of proliferation, we added RA or AGN at different time points. The results show that RA reduced mineralization even when added as late as day 14 (21%), after completion of the proliferation phase. Moreover, the RAR antagonist increased mineralization (16%) also when added after completion of the proliferation phase (Fig. 3C). Together, these *in vitro* findings are in agreement with the dynamic histomorphometric results described above.

### RA reduces *Alpl* and *Bglap* (*Osteocalcin)* expression

**Figure 4:**
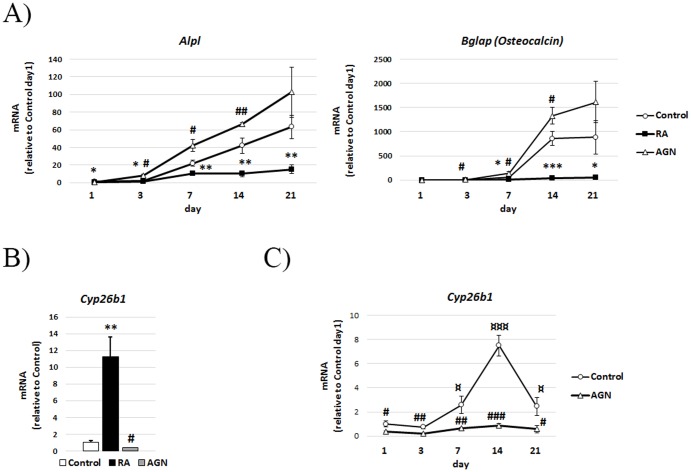
QRT-PCR analysis of genes associated with osteoblast differentiation and endogenous RA degradation. (**A**) Expression levels of mRNA for *Alpl (alkaline phosphatase, liver/bone/kidney)* and *Bglap* (*Osteocalcin)* during a mineralization experiment of MC3T3-E1 cells treated with 400 nM RA or 1 µM AGN. (**B**) mRNA expression of *Cyp26b1* at day 1, treated as in (A). (**C**) mRNA expression of *Cyp26b1* at day 1, 3, 7, 14 and 21 of a mineralization experiment of MC3T3-E1 cells +/– 1 µM AGN. Means +/– SD, * p<0.05, ** p<0.01 and *** p<0.001 RA vs Control. # p<0.05, ## p<0.01 and ### p<0.001 AGN vs Control. ¤ p<0.05 and ¤¤¤ p<0.001 vs Control day 1.

Quantitative Real Time-PCR (QRT-PCR) analysis of MC3T3-E1 cells at different time points during mineralization demonstrated a continuous up-regulation of the osteoblast marker *alkaline phosphatase, liver/bone/kidney (Alpl)* up to day 21 in control cells (Fig. 4A). *Bglap* (*Osteocalcin)*, a marker of mature osteoblasts, shows a distinct increase from day 7. In line with the Alizarin data, addition of RA inhibits the induction of *Alpl* and *Bglap (Osteocalcin)* expression. Addition of the RAR antagonist increased *Alpl* and *Bglap (Osteocalcin)* expression levels above controls from day 3 (Fig. 4A).

### Cyp26b1 is induced during normal osteoblast differentiation

As expected RA quickly and distinctly increased expression of the RA degrading enzyme *Cyp26b1* in MC3T3-E1 cultures after 1 day (Fig. 4B). During normal osteoblast differentiation *Cyp26b1* expression began to rise at day 7 and increased distinctly to day 14 followed by a drop in expression on day 21 (Fig. 4C). Presence of the RAR antagonist suppressed the expression of *Cyp26b1* significantly at all time points as expected, since *Cyp26b1* is a known direct RA target gene (Fig. 4C).

### RA reduces Runx2 and Osterix protein levels

**Figure 5:**
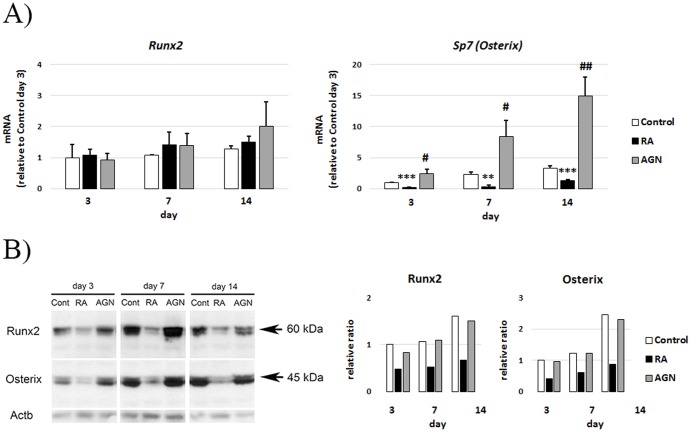
Runx2 and Sp7 (Osterix) levels in RA and RAR antagonist treated MC3T3-E1 cells. (**A**) QRT-PCR analysis of *Runx2* and Sp7 (*Osterix)* expression at day 3, 7 and 14 of cells treated with 400 nM RA or 1 µM AGN. (**B**) Representative Western blot analysis of Runx2 and Osterix at day 3, 7 and 14 of cells treated as in (**A**) and quantification of the Western bands relative to Actb (relative ratio). Means +/– SD, ** p<0.01 and *** p<0.001, RA vs Control, and ^#^ p<0.05, ^##^ p<0.01 AGN vs Control.

The efficient inhibition of *Bglap (Osteocalcin)* expression by RA indicates that RA interferes with pivotal transcription factors during early osteoblast commitment. To examine this possibility we analyzed for expression of *Runx2* and *Sp7* (*Osterix)*, two key osteoblast transcription factors necessary for osteoblast differentiation and mineralization. QRT-PCR analysis of MC3T3-E1 cells taken at day 3, 7 and 14 from mineralization experiments showed that *Runx2* transcript levels are not significantly regulated by differentiation, RA or AGN (Fig. 5A). In contrast, *Sp7* (*Osterix)* levels were markedly suppressed by RA treatment already at day 3 (Fig. 5A). In addition, AGN treatment increased *Sp7 (Osterix)* transcript levels at all time points measured. Opposite to the transcript levels, Runx2 protein levels increased in the control (Fig. 5B). RA treatment reduced Runx2 protein levels from day 3 (Fig. 5B). Similarly to Runx2, Osterix protein levels clearly decreased in RA treated cells (Fig. 5B).

### RA reduces Phex in a RAR-dependent way

**Figure 6:**
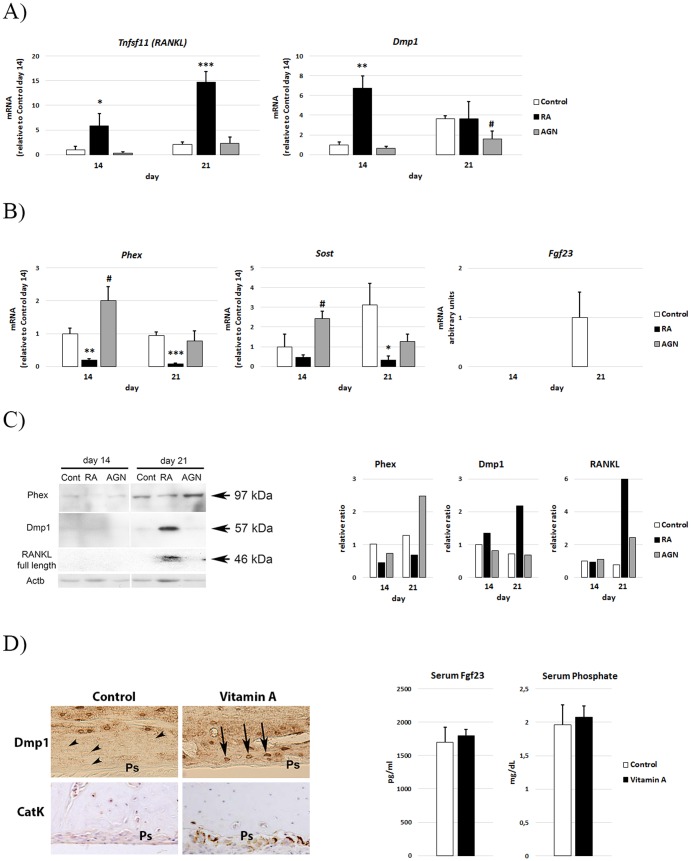
Osteocyte markers in RA and RAR antagonist treated MC3T3-E1 cells and periosteal and serum phenotype in vitamin A treated rats. QRT-PCR analysis of *Tnfsf11 (RANKL)* and *Dmp1,* (**A**) *Phex, Sost* and *Fgf23* (**B**) expression at day 14 and 21 during a mineralization experiment of MC3T3-E1 cells treated with 400 nM RA or 1 µM AGN. (**C**) Representative Western blot analysis of Phex, Dmp1 and full length RANKL at day 14 and 21 of MC3T3-E1 cells treated as in (A) and quantification of Western bands relative to Actb (relative ratio). (**D**) Dmp1 and Cathepsin K (CatK) immunohistochemical staining at the diaphyseal periosteal site in rats suffering from hypervitaminosis A and in control rats. Upper panel: Arrow heads indicate Dmp1 negative osteocytes close to the periosteum (Ps) in control rat bone and arrows indicate Dmp1 positive osteocytes close to the periosteum in hypervitaminosis A rat bone. Lower panel: only Vitamin A animals show clear CatK staining at the Ps site. (**E**) Serum Fgf23 and phosphate levels in rats from (D). Means +/– SD, * p<0.05, ** p<0.01 and *** p<0.001 RA vs Control. ^#^ p<0.05, ^##^ p<0.01 AGN vs Control.

We then investigated how RA affects the expression of genes associated with a late osteoblast/osteocyte phenotype. As shown in Fig 6A, control MC3T3-E1 cells showed increased transcripts of *Tnfsf11* (*RANKL)* and *Dmp1* at day 21 compared to day 14. RA treatment induced transcripts of both *Tnfsf11 (RANKL)* and *Dmp1* at day 14, and by day 21 *Tnfsf11 (RANKL)* had increased 7-fold above control. In contrast, RA treatment prevented induction of *Phex, Sost* and *Fgf23* (Fig. 6B). In line with a negative effect of RAR signaling on *Phex* transcription, AGN treatment caused a significant increase in *Phex* transcript levels at day 14 (Fig. 6B). Consistent with the QRT-PCR results, immunoblot analysis demonstrated that RA decreased and AGN increased Phex protein levels (Fig. 6C). Finally, RA treatment of MC3T3-E1 osteoblasts induced a concomitant increase of Dmp1 and full-length RANKL protein at day 21 (Fig. 6C). In agreement with this finding *in vitro*, a high vitamin A intake markedly increased osteocytic Dmp1 and osteoclastic cathepsin K staining at the periosteal site at the diaphysis *in vivo* (Fig. 6D), the site of the reduced mineralization apposition rate (Fig. 1B). Neither serum Fgf23 levels nor serum phosphate levels were significantly different in the rats suffering from hypervitaminosis A compared to controls (Fig. 6E).

## Discussion

By using pair-feeding to control for indirect effects on appetite and general growth, we could show that a high dietary vitamin A intake has a direct, negative effect on bone mineralization and diaphyseal radial growth in rats. In accordance with this *in vivo* observation, RA also inhibited mineralization *in vitro*. This appeared mainly to be a consequence of an inhibition of induction of the key transcription factors Runx2 and Osterix. In addition, Phex, which is necessary for normal mineralization, was suppressed in RA treated cells.

It has been shown that rats and mice, irrespectively of age and sex, respond rapidly to hypervitaminosis A with a reduced bone diameter, however the magnitude of the response is lower in skeletally mature animals and slower with lower doses [5], [6]. In the present study we have mimicked early hypervitaminosis A protocols using high doses of retinol in young male rats to induce distinct changes in a short time. The serum vitamin A level in these rats was similar to that seen in hypervitaminosis A toxicity in humans [25].

Interestingly, normal osteoblast differentiation involved up-regulation of *Cyp26b1,* the major enzyme responsible for RA degradation, suggesting that a drop in RA signaling is required for osteoblast mineralization analogous to what has been found for chondrogenesis [26], [27]. In line with this, we showed that addition of a Cyp26 inhibitor led to reduced mineralization. The negative effect of endogenous RA on osteoblast mineralization was further demonstrated by the finding that blocking RAR-signaling with a pan-RAR antagonist increased mineralization. This together with the recent observations that all three RAR receptors are downregulated during osteoblast differentiation support the view that RAR signaling is a negative regulator of osteoblast mineralization [28]. Previous studies on the effects of exogenous RA on mineralizing cultures of osteoblasts are consistent with our results [6], [29]–[32] except one study which used a very high, supraphysiological concentration of RA (10 µM) [33]. When AGN was added together with RA, AGN completely reversed the effect of RA in the primary osteoblast cultures whereas it only partially blocked the negative effect of RA on mineralization in MC3T3-E1 cells. A reason for this difference might arise from that preparations of primary osteoblasts consist of a mix of osteoblasts and non-osteoblastic cells. In the MC3T3-E1 cells, AGN treatment consistently and significantly increased Alizarin staining although the magnitude varied. This may be due to a combination of extensive plasticity of mature osteoblasts together with the long culturing times [34], [35].

*In vivo*, both humans and mice lacking Cyp26b1 show reduced ossification of calvarial bones, which is consistent with our findings in rat periosteal bone and the MC3T3-E1 cell line from mouse calvaria. However, these mutants also have vertebral, joint, and cranial bone fusions [13], [36]–[40]. This may seem contradictory, but in our opinion the defect behind these fusions is more likely to be morphogenic than a direct consequence of increased osteoblast mineralization. It may also involve non-osteoblastic cells since it is well-known that chondrocytes or pluripotent cells, such as embryonic stem cells, preadipocytes, adipose-derived adult stromal cells and adipose-derived mesenchymal cells, respond to RA by enhanced mineralization [41]–[47]. Along these lines, and specifically regarding the observed craniosynostosis in Cyp26b1 mutants, it has been shown that cranial suture-derived mesenchymal cells enhance osteogenesis upon RA treatment [48]. The mechanism behind these opposite functions of RA in osteoblasts and non-osteoblastic cells is unclear, but may involve the bzip transcription factor CCAAT/Enhancer Binding Protein beta, which showed opposite effects of RA on Runx2 expression in mesenchymal stem cells and committed osteoblasts [49].

Our study showed a distinct reduction of Runx2 protein upon RA treatment, whereas Runx2 transcript levels were unchanged, consistent with several studies that have indicated that the Runx2 transcript levels are less clearly regulated [50]–[53]. It is not yet known how RA alters Runx2 protein levels, but one possible mechanism could be via RA-induced proteasomal degradation, in analogy to how RA induces degradation of phosphorylated Smad1 [54]. Alternatively, the decreased Osterix protein levels may result in reduced Runx2 protein stability, as it was recently shown that Runx2 and Osterix protein co-expression synergistically increases their stability [55].

That vitamin A is a negative regulator of bone mineralization is further strengthen by the recent observation that a retinol-free diet is more efficient than a high phosphate diet in rescuing the mineralization defects in the Phex mutant Hyp mouse [56]. The underlying cause of the reduced mineralization in the Hyp mouse is the inactivation of the Phex gene. Phex has been shown to function in concert with Dmp1 and Fgf23 in regulating phosphate metabolism [57]. Mice lacking both Phex and Dmp1 have non-additive effects, suggesting a common pathway for these proteins [58]. Notably, a Dmp1 transgene does not rescue but instead further reduces bone mineralization in Hyp mice. This indicates that in the presence of low Phex levels, Dmp1 has a negative effect of bone mineralization [58]. Since RA downregulated Phex and upregulated Dmp1 levels in our *in vitro* cultures, this may, in part, have contributed to the reduced mineralization. Furthermore, RA prevented the induction of Fgf23 at the osteoblast level which was not reflected at the serum level, but is in line with the fact that retinol deprivation appears to impact the cell-autonomous mineralization defect of Hyp osteoblasts but not serum parameters [56].

Previously published data suggest that RA can induce an osteocytic phenotype in short- and long-term cultures [13], [59]. Here, using 400 nM of RA during long-term culture (weeks), we observed that the expression of the osteocyte-associated genes RANKL and Dmp1 increased (which has also been observed earlier by us and others [13], [60]). In contrast, other osteocyte-associated genes such as *Phex*, *Sost* and *Fgf23* were downregulated, which is opposite to recent studies [13], [59]. The explanation for these discrepancies is probably that Laue et al. [13] used a higher concentration of RA, fasting MCT3T-E1 cells and much shorter treatment time (48 h), and that Mattinzoli el al. [59] used a very high (10 µM) concentration of RA. In this context it is worth pointing out that induction of osteocyte markers in cell culture takes quite a long time, e.g. it takes 14 days to produce detectable amounts of the osteocyte-specific Sost protein in the osteocyte cell line IDG-SW3 [61]. We showed here that the RANKL and Dmp1 proteins first appeared clearly on day 21 in the RA treated preosteoblastic cell line, MC3T3-E1. The concomitant expression of Dmp1 and full-length RANKL induced by RA *in vitro* is in agreement with our present and previous [19] *in vivo* observations of increased periosteal Dmp1 staining and the number of cathepsin K-positive osteoclasts in hypervitaminosis A. Although we have not been able to stain for full-length RANKL in these bone sections, it is tempting to speculate that vitamin A concomitantly induces Dmp1 and RANKL also *in vivo*, and that the full-length RANKL in these periosteal cells stimulates the formation of periosteal osteoclasts. Further studies will be needed to clarify this.
